# Accurate detection of early-stage lung cancer using a panel of circulating cell-free DNA methylation biomarkers

**DOI:** 10.1186/s40364-023-00486-5

**Published:** 2023-04-26

**Authors:** Shuo Hu, Jinsheng Tao, Minhua Peng, Zhujia Ye, Zhiwei Chen, Haisheng Chen, Haifeng Yu, Bo Wang, Jian-Bing Fan, Bin Ni

**Affiliations:** 1 Department of Thoracic Surgery, The First Affiliated Hospital of Soochow University, Suzhou, China; 2 Anchordx Medical Co., Ltd, Guangzhou, China; 3 AnchorDx Inc, Fremont, CA USA; 4 Haian People’s Hospital, Haian, China; 5 The Fifth People’s Hospital of Wuxi, Wuxi, China; 6 Department of Pathology, Southern Medical University, Guangzhou, China

**Keywords:** DNA methylation, Differential methylated region, Early diagnosis, Lung cancer, Noninvasive

## Abstract

**Background:**

Lung cancer remains the leading cause of cancer mortality worldwide. Early detection of lung cancer helps improve treatment and survival. Numerous aberrant DNA methylations have been reported in early-stage lung cancer. Here, we sought to identify novel DNA methylation biomarkers that could potentially be used for noninvasive early diagnosis of lung cancers.

**Methods:**

This prospective-specimen collection and retrospective-blinded-evaluation trial enrolled a total of 317 participants (198 tissues and 119 plasmas) comprising healthy controls, patients with lung cancer and benign disease between January 2020 and December 2021. Tissue and plasma samples were subjected to targeted bisulfite sequencing with a lung cancer specific panel targeting 9,307 differential methylation regions (DMRs). DMRs associated with lung cancer were identified by comparing the methylation profiles of tissue samples from patients with lung cancer and benign disease. Markers were selected with minimum redundancy and maximum relevance algorithm. A prediction model for lung cancer diagnosis was built through logistic regression algorithm and validated independently in tissue samples. Furthermore, the performance of this developed model was evaluated in a set of plasma cell-free DNA (cfDNA) samples.

**Results:**

We identified 7 DMRs corresponding to 7 differentially methylated genes (DMGs) including HOXB4, HOXA7, HOXD8, ITGA4, ZNF808, PTGER4, and B3GNTL1 that were highly associated with lung cancer by comparing the methylation profiles of lung cancer and benign nodule tissue. Based on the 7-DMR biomarker panel, we developed a new diagnostic model in tissue samples, termed “7-DMR model”, to distinguish lung cancers from benign diseases, achieving AUCs of 0.97 (95%CI: 0.93-1.00)/0.96 (0.92-1.00), sensitivities of 0.89 (0.82–0.95)/0.92 (0.86–0.98), specificities of 0.94 (0.89–0.99)/1.00 (1.00–1.00), and accuracies of 0.90 (0.84–0.96)/0.94 (0.89–0.99) in the discovery cohort (n = 96) and the independent validation cohort (n = 81), respectively. Furthermore, the 7-DMR model was applied to noninvasive discrimination of lung cancers and non-lung cancers including benign lung diseases and healthy controls in an independent validation cohort of plasma samples (n = 106), yielding an AUC of 0.94 (0.86-1.00), sensitivity of 0.81 (0.73–0.88), specificity of 0.98 (0.95-1.00), and accuracy of 0.93 (0.89–0.98).

**Conclusion:**

The 7 novel DMRs could be promising methylation biomarkers that merits further development as a noninvasive test for early detection of lung cancer.

**Graphical abstract:**

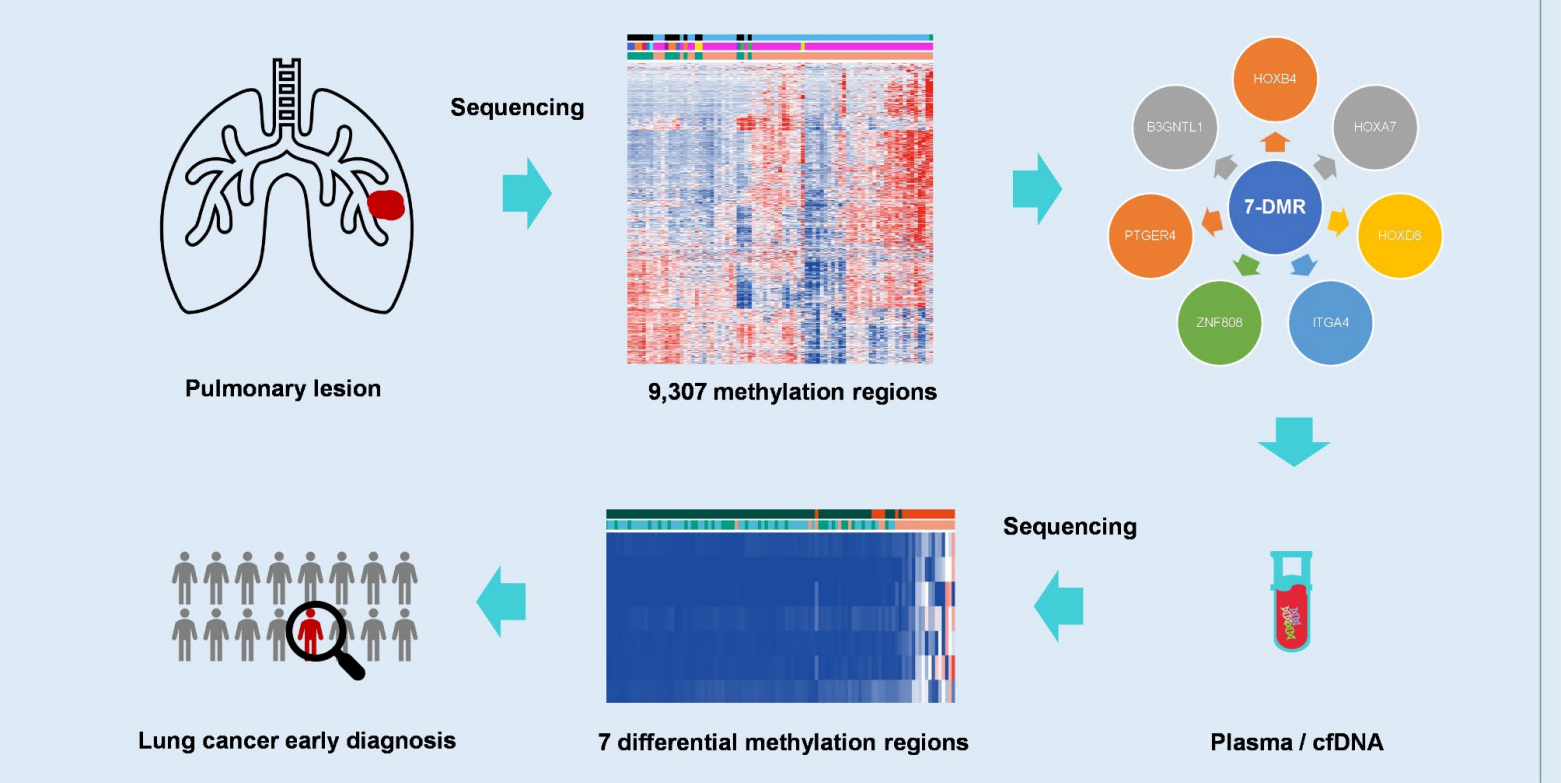

**Supplementary Information:**

The online version contains supplementary material available at 10.1186/s40364-023-00486-5.

## Introduction

Lung cancer remains the leading cause of cancer mortality worldwide so far. In 2020, 2.2 million new cases were found globally and 1.8 million were dead, representing 18.0% of all cancer deaths [1]. Data suggest that around 68-92% of patients survive at least 5 years when diagnosed at the earliest stage, but this falls to just 10% for those diagnosed with the most advanced disease (stage IV) [2]. In most people, the cancer has already spread beyond its original site to a distant part of the body by the time they have symptoms and seek medical care. Early detection of lung cancer helps to improve treatment and survival [3].

Low-dose Computed Tomography (CT) is commonly used for detection of pulmonary nodules, but the ambiguous risk evaluation often causes overdiagnosis. Numerous antigens in the blood have been investigated for years as potential biomarkers of lung cancer. The most intensively studied biomarkers include cytokeratin 19 fragment (CYFRA 21 − 1) [4], carcinoembryonic antigen (CEA) [5], neuron specific enolase (NSE) [6], and squamous cell carcinoma antigen (SCC-Ag) [7]. But the performances of those biomarkers for early diagnosis are unsatisfactory due to the low sensitivity. Accordingly, it is highly desirable to find effective and specific diagnostic biomarkers for early-stage lung cancer.

Plenty of studies have shown that DNA methylation is strongly related to the occurrence and progression of various tumors [8]. DNA methylation is an epigenetic modification of genes involving the covalent transfer of S-adenosylmethionine as methyl group donor to the C-5 position of the cytosine ring of DNA to form 5-methylcytosine by catalysis of DNA methyltransferases [9]. According to the reported studies, extensive DNA hypomethylation was found in the whole genome of tumor cells, leading to the activation of proto-oncogenes and increased genomic instability [10]. The methylation status of tumor cells in the promoter regions of tumor suppressor genes and repair genes is increased, that is, hypermethylation, which leads to the inhibition of the expression of corresponding tumor suppressor genes [11, 12]. The hypermethylated genes of tumor cells mostly occur in CpG islands in the promoter region, while the CpG islands in the promoter region of normal cells are mostly in a non-methylated state [8]. Aberrations in DNA methylation are found at the genomic level in most tumors, including lung cancer, as well as in patients with non-neoplastic diseases such as Alzheimer’s disease and heart failure [13, 14]. Many studies have found that different diseases and even different stages of a disease may have specific methylation patterns [15]. The frequency of CpG island hypermethylation in tumor cells is much higher than that of gene mutation [16]. Therefore, by detecting the methylation level of a specific set of genes or the whole genome, it is possible to predict the risk of lung cancer [17–19].

In this study, based on our DNA methylation sequencing data, we identified seven novel methylation biomarkers by comparing the methylation profiles of tissue samples from lung cancer and benign lung disease for early diagnosis of lung cancer. Based on the 7-DMR biomarker panel, we constructed a new diagnostic model that could predict the malignant risk of lung cancer based on blood samples and could be further developed as a noninvasive diagnostic test.

## Methods

### Participating patients and Sample Collection

A total of 317 subjects were recruited, including 50 healthy controls with matched age and gender, and 267 patients with lung nodule indicated by CT/LDCT scan at The First Affiliated Hospital of Soochow University in China from Jan 2020 to Dec 2021. All enrolled patients with lung diseases were at high risk of lung cancer and thus had undergone surgical resection. None of patients received any preoperative cancer therapies. 10 mL of peripheral blood was collected from eligible patients 1–3 days prior to surgical operation. Formalin-fixed paraffin embedded (FFPE) tissue samples were obtained from subsequently surgical resections. Pathological information of all samples was determined based on surgically resected tissue sections according to 2015 WHO Histological Classification of Lung Cancer [20]. Written informed consents were provided by all participants. This study was approved by the Ethical Committees of The First Affiliated Hospital of Soochow University.

As additional detail on methods of **tissue/blood sample processing, targeted cell-free DNA methylation sequencing, and sequencing data analysis** are provided in an online data supplement.

### Differential methylation analysis

Differential methylation analysis was conducted using R package DSS (version 2.14.0) [21]. Differentially methylated CpGs (DMCs) were first identified (criteria: FDR < 0.05, delta > 0.05), and then adjacent DMCs were merged into DMRs. The DMR required at least 3 CpG sites and the distance between nearby CpG sites was not more than 100 base pairs. DMRs were intersected with protein-coding genes (hg19 Ensembl (v75), n = 20,232) by using annovar [22].

### Unsupervised hierarchical clustering of DNA methylation profiles

The methylation profiles of tissue samples in discovery cohort and independent validation cohort obtained with our custom-made methylation panel consisting of 9307 informative lung cancer DMRs were used for unsupervised hierarchical clustering. The methylation level of each targeted regions was calculated as the ratio of the methylated CpGs and the total sequenced CpGs (sum of methylated and unmethylated CpGs). Before clustering, the methylation level of each targeted region was Z-score normalized. To calculate the Z-score of each targeted region for each sample, we subtracted its mean from each of the samples and divided the result by its standard deviation. The R function “hclust” was used to perform hierarchical clustering with “ward.D2” as the clustering algorithm. R package pheatmap was used to plot the heat map after hierarchical clustering.

### Diagnostic marker selection

The machine learning task was conducted with the intention to identify a DNA methylation biomarker panel for accurate diagnosis of early-stage lung cancer. We first filtered DMRs for a maximum of 30% of coefficient of variation calculated from analytical replicates (quality assessment samples) to ensure good analytical reproducibility of the selected DMRs. We then performed marker selection using a Python implementation (https://github.com/smazzanti/mrmr) of the minimum redundancy and maximum relevance (mRMR) feature selection algorithm. We examined the relationship between model performance and number of features (from 1 to 10) based on fivefold, ten times cross-validation in the discovery cohort. We limited the maximum number of features to 10 out of practical considerations: a marker panel based on a relatively small set of DMRs may be easier to translate and implement into clinical practice. We then determined the optimal number of features (or marker panel) according to the ‘maximal AUC score using the minimal set of DMRs’ principle.

### Gene Ontology Enrichment Analysis and Pathway Enrichment analyzes for diagnostic biomarkers

KEGG (Kyoto Encyclopedia of Genes and Genomes) pathway analysis and GO (Gene Ontology) pathway analysis were conducted by the clusterProfiler R package.

### Analysis of gene expression with TCGA data

Gene expression data were downloaded from the cancer genome atlas (TCGA) database (https://portal.gdc.cancer.gov/). Gene expression data of Lung Adenocarcinoma (LUAD) (59 normal, 535 cancer) and Lung Squamous Cell Carcinoma (LUSC) (49 normal, 502 cancer) cancer tissues and adjacent tissues were collected. The gene expressions of HOXB4, B3GNTL1, ZNF808, HOXD8, ITGA4, PTGER4, and HOXA7 were compared between lung cancer tissue and adjacent normal tissue.

### Performance evaluation of 7 candidate diagnostic biomarkers in TCGA data

DNA methylation datasets in which methylation level of each CpG site was denoted by beta value were retrieved from the cancer genome atlas (TCGA) database (https://portal.gdc.cancer.gov/). The DNA methylation level of HOXB4, B3GNTL1, ZNF808, HOXD8, ITGA4, PTGER4, and HOXA7 in Lung Adenocarcinoma (31 normal, 473 cancer) and Lung Squamous Cell Carcinoma (42 normal, 370 cancer) cancer tissues and adjacent tissues were collected.

### Construction of 7-DMR model

First, to evaluate diagnostic performance of the 7-DMR methylation panel for classifying lung cancer tissue samples, a predictive model was developed by fitting a logistic regression model using the 7 DMRs in the discovery cohort as the input. Python’s scikit-learn package (v0·20·0) was used to perform the logistic regression with default parameters: penalty = l2, tol = 1e-4, C = 1.0, fit_intercept = True, class_weight = None, solver = lbfgs, max_iter = 100. Then, to test the discriminative power of the 7-DMR methylation panel for the noninvasive detection of early-stage lung cancer with cell-free DNA from plasma samples, all tissue samples were pooled to construct a predictive model with logistic regression. The model was then applied to a plasma cohort consisting of patients with lung cancer and benign diseases, as well as healthy controls.

### Statistical analyses

All statistical analyses were conducted using R software (v3.32). Continuous variables were presented as means and standard deviations or medians and ranges, while categorical variables were presented as whole numbers. Continuous variables were compared using Student’s t test, while categorical variables were compared using the chi-square test. 95% confidence intervals (CI) for AUC, sensitivity, specificity, accuracy of the models was calculated using a binomial distribution. Receiver operating characteristic (ROC) analysis was performed using the pROC R package (v1.15.3). Unless otherwise specified, all statistical tests were conducted using a two-sided alpha level of 0.05.

## Results

### Clinical cohorts

**Fig. 1:**
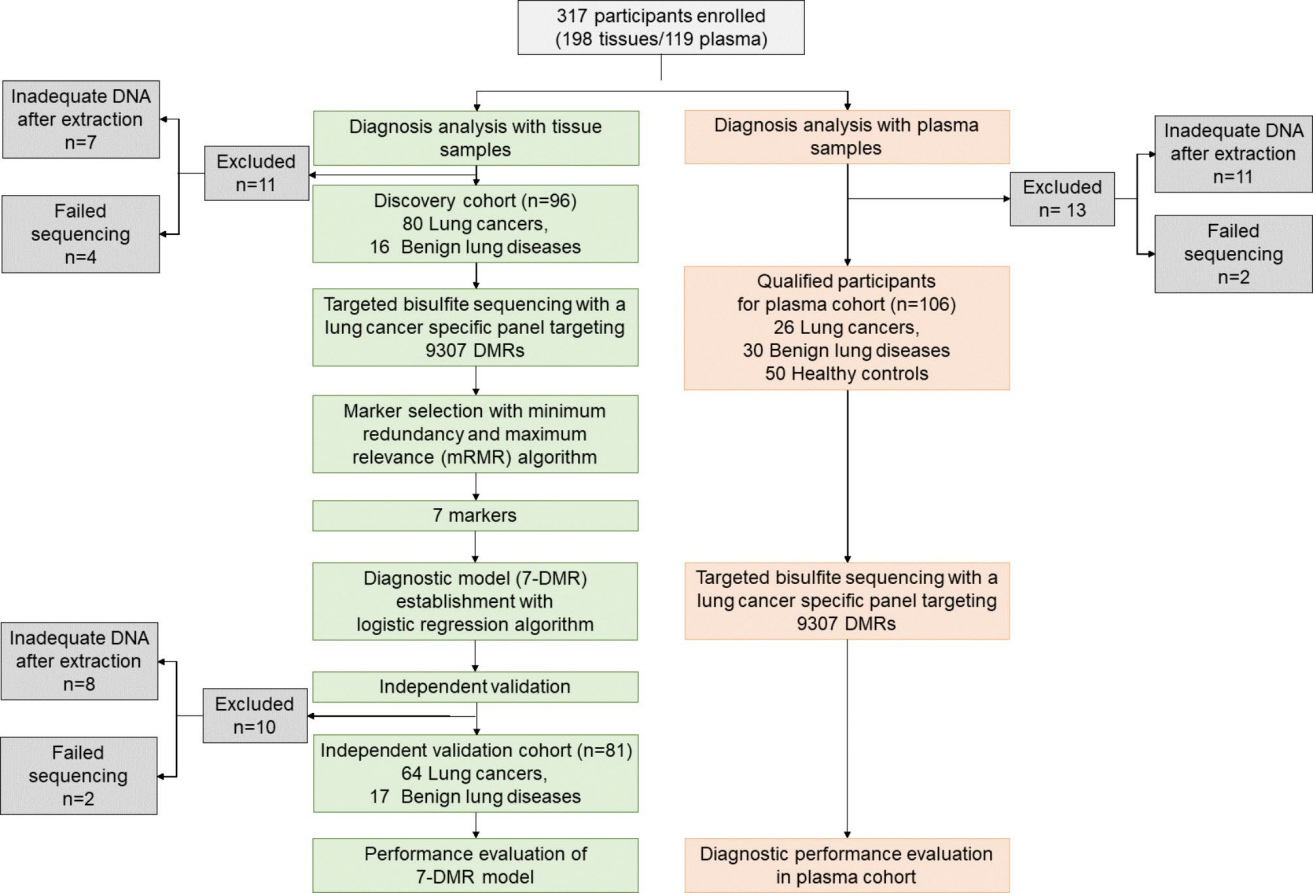
**Flowchart for finding lung cancer candidate diagnostic biomarkers.** Total 317 patients enrolled. 7-DMR model was developed on 96 tissue samples and independently validated on 81 tissue samples. The noninvasive diagnostic performance of 7-DMR model was evaluated in 106 plasma samples.

We collected a total of 198 tissue samples that were used to find differential DNA methylation biomarkers for early diagnosis of lung cancer. 21 subjects were excluded due to inadequate DNA after extraction (n = 15) and failed sequencing (n = 6). Consequently, 96 samples (80 lung cancers, 16 benign lung diseases) were used for 7-DMR model discovery cohort and the remaining 81 samples (64 lung cancers, 17 benign diseases) were used as an independent validation cohort. In total, 119 plasma samples were collected to evaluate the performance of the diagnostic model. 13 were excluded due to inadequate DNA or failed sequencing and the rest 106 subjects (26 lung cancers, 30 benign diseases, 50 healthy controls) were included for subsequent analysis (Fig. 1). There was no statistically significant difference in age among all three cohorts. The cohorts contained 88.2% of early-stage patients (stages 0/I) for identifying features correlated with early-stage lung cancer. 71.4% of all were never smokers. The patient demographic and clinical characteristics were summarized in Table 1.

**Table 1:** Patient Demographic and Clinical Characteristics

Characteristics	Patients, No. (%)			
	Discovery cohort	Validation cohort	Plasma cohort	Overall
Total, n	N = 96	N = 81	N = 106	N = 283
Age-year				
Mean (SD)	54.7 (11.0)	54.2 (9.48)	54.6 (10.1)	54.5 (10.2)
Median [Min, Max]	56.0 [28.0, 81.0]	53.0 [33.0, 79.0]	55.0 [21.0, 83.0]	55.0 [21.0, 83.0]
Gender				
Male	46 (47.9)	38 (46.9)	65 (61.3)	149 (52.7)
Female	50 (52.1)	43 (53.1)	41 (38.7)	134 (47.3)
Smoking_history				
Never smoker	75 (78.1)	59 (72.8)	68 (64.2)	202 (71.4)
Smoker	21 (21.9)	22 (27.2)	38 (35.8)	81 (28.6)
Family history of lung cancer				
No	88 (91.7)	77 (95.1)	103 (97.2)	268 (94.7)
Yes	8 (8.3)	4 (4.9)	3 (2.8)	15 (5.3)
Histopathology				
Lung cancer	80 (83.3)	64 (79.0)	26 (24.5)	170 (60.1)
Lung adenocarcinoma	78 (81.3)	63 (77.8)	23 (21.7)	164 (58.0)
Squamous cell carcinoma	1 (1.0)	0 (0)	3 (2.8)	4 (1.4)
Large cell carcinoma	1 (1.0)	1 (1.2)	0 (0)	2 (0.7)
Benign disease	16 (16.7)	17 (21.0)	30 (28.3)	63 (22.3)
Inflammation	6 (6.3)	5 (6.2)	16 (15.1)	27 (9.5)
Pulmonary fibrosis	3 (3.1)	5 (6.2)	5 (4.7)	13 (4.6)
Hamartoma	0 (0)	2 (2.5)	4 (3.8)	6 (2.1)
Pulmonary sclerosing pneumocytoma	1 (1.0)	1 (1.2)	4 (3.8)	6 (2.1)
Tuberculosis	3 (3.1)	1 (1.2)	0 (0)	4 (1.4)
Fungal infection	2 (2.1)	2 (2.5)	1 (0.9)	5 (1.8)
Atypical adenomatous hyperplasia	1 (1.0)	1 (1.2)	0 (0)	2 (0.7)
Healthy control	0 (0)	0 (0)	50 (47.2)	50 (17.7)
Stage				
0	1 (1.3)	0 (0)	0 (0)	1 (0.6)
I	76 (95.0)	63 (98.4)	10 (38.5)	149 (87.6)
II	1 (1.3)	1 (1.6)	8 (30.8)	10 (5.9)
III	2 (2.5)	0 (0)	4 (15.4)	6 (3.5)
IV	0 (0)	0 (0)	4 (15.4)	4 (2.4)

### Identification of differential methylation regions for lung cancer diagnosis

**Fig. 2:**
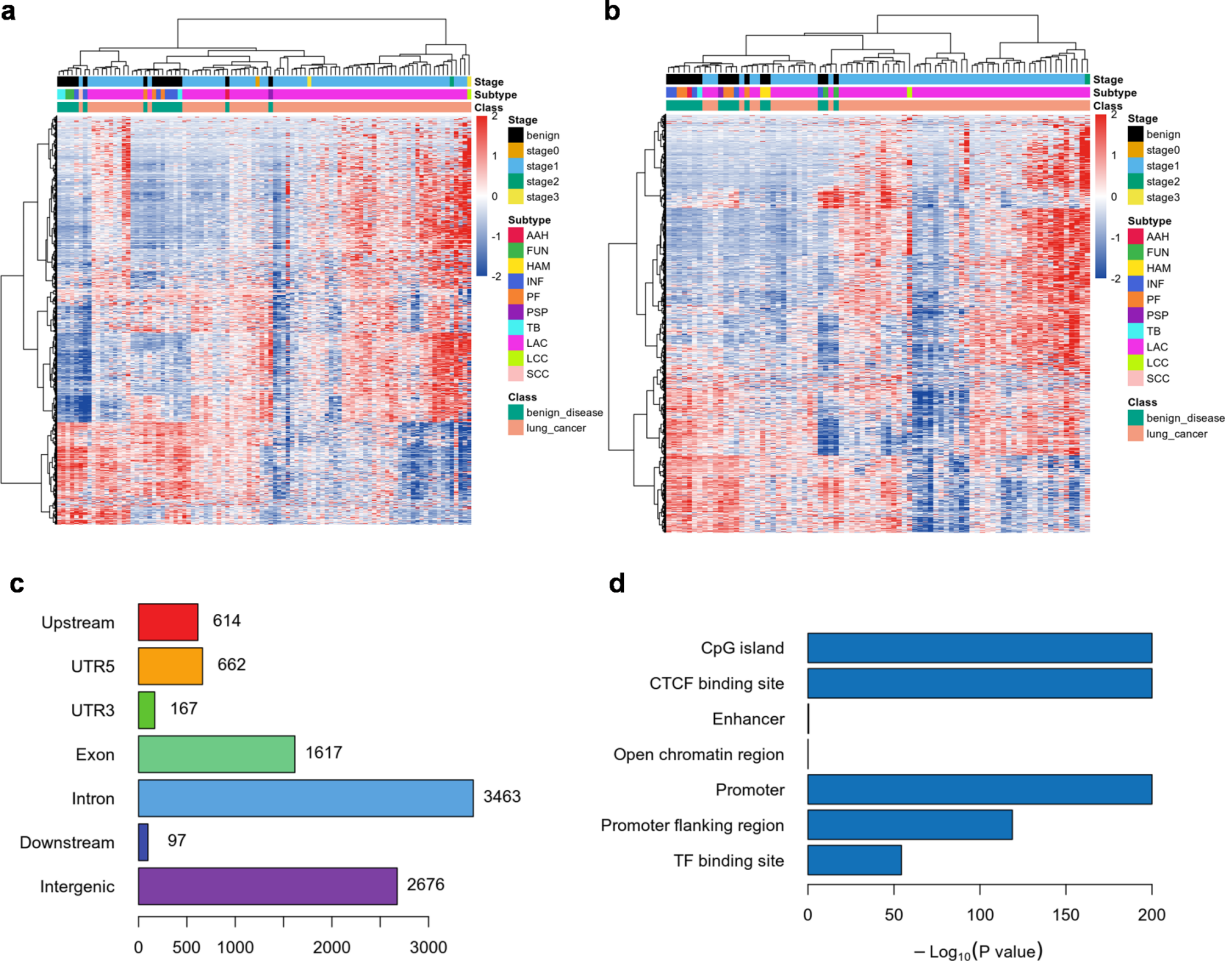
**Identifying lung cancer-specific differentially methylated regions. (a-b)** Heatmap of the differentially methylated sites in lung cancer and benign pulmonary nodule tissues in training dataset **(a)** and validation dataset **(b)**, contains 6604 hypermethylated and 2703 hypomethylated regions. **(c)** The region on the genes where the hypermethylated and hypomethylated sites are located. **(d)** The correlation between hypermethylated and hypomethylated DMRs and regulatory regions in genome.

By comparing the methylation profiles of tissue samples between lung cancer and benign lung disease, 6604 hypermethylated and 2703 hypomethylated DMRs were found (Fig. 2a-b), corresponding to 2614 hypermethylation and 1228 hypomethylated genes. The abnormal methylation regions were predominantly located at the intron, intergenic, and exon regions (Fig. 2c), and highly enriched in CpG island, promoter, CTCF binding site, promoter flanking region, and TF binding site (Fig. 2d). This was consistent with the general characteristics of aberrant DNA methylation in solid tumors. KEGG pathway analysis indicated that the following pathways were closely associated with the genes: Regulation of actin cytoskeleton, Non-small cell lung cancer, Wnt signaling pathway, Axon guidance, Hippo signaling pathway, etc. (Figure S1). Meanwhile, GO enrichment pathway analysis shown that a variety of cellular components, molecular functions and biological processes may be involved, especially the axon part, DNA-binding transcription activator activity, and embryonic organ morphogenesis (Figure S2a-c).

### Identification of candidate methylated biomarkers for lung cancer diagnosis

**Fig. 3:**
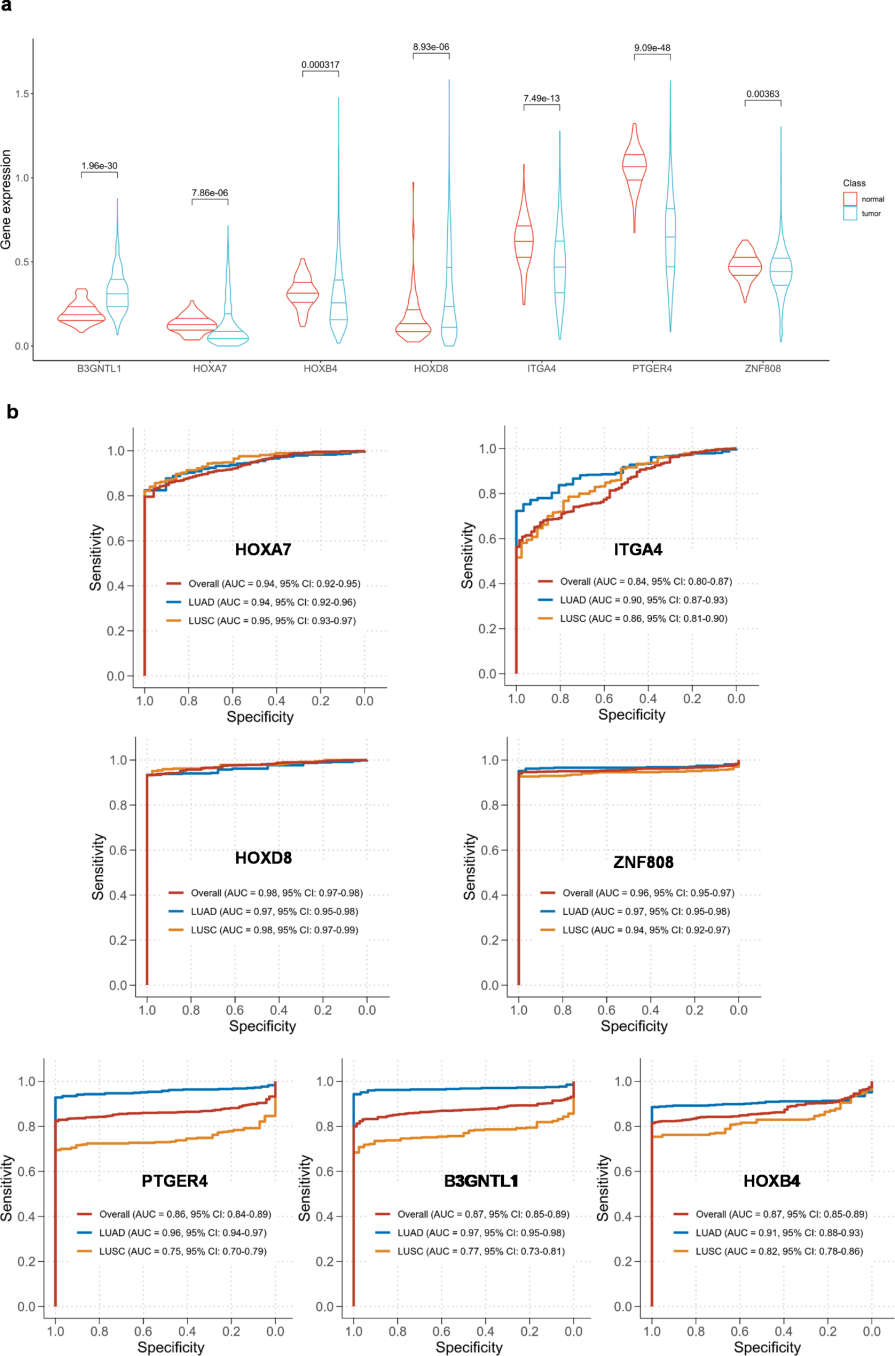
**Gene expression and diagnostic performance of the 7 DMRs in TCGA. (a)** The comparison of gene expression levels between lung cancer and normal controls based on TCGA data. **(b)** The representative ROC curves display the classification performance of each DMR in LUAD/LUSC vs. normal based on TCGA data.

Then we used the minimum redundancy and maximum relevance (mRMR) algorithm to assess the predictive power of each DMR and finally selected the most significant 7 DMRs: chr17:46655603–46,655,750, chr7:27195684–27,195,794, chr2:176993563–176,993,743, chr2:182322423–182,322,574, chr19:53038958–53,039,010, chr5:40681077–40,681,250, and chr17:80943984–80,944,093. The genes corresponding to these 7 methylation regions were HOXB4, HOXA7, HOXD8, ITGA4, ZNF808, PTGER4, and B3GNTL1 (Table 2). To investigate the correlation between the seven differential methylation regions and progression of lung cancer, we compared the expression of these seven corresponding genes in lung cancer and normal controls based on TCGA gene expression data, which contained LUAD (59 normal, 535 cancer) and LUSC (49 normal, 502 cancer). It turned out that the expressions of B3GNTL1 and HOXD8 were significantly upregulated, while the expression of remaining five genes (HOXB4, ZNF808, ITGA4, PTGER4, and HOXA7) were significantly downregulated in lung cancer tissues (p < 0.01) (Fig. 3a). This revealed the potential biological and clinical significance of the seven genes in the formation of lung cancer.

To test the diagnostic capabilities of seven markers in distinguishing between lung cancer and normal controls, we analyzed the performance of seven DMRs individually based on TCGA database that included LUADs (31 normal, 473 cancer) and LUSCs (42 normal, 370 cancer). Regarding the LUAD, all the 7 markers achieved AUCs varied from 0.90 to 0.97. While for the LUSC, the models of PTGER4 and B3GNTL1 reached AUCs of 0.75 (95%CI: 0.70–0.79) and 0.77 (0.73–0.81), respectively. ITGA4 and HOXB4 yielded AUCs of 0.86 (0.81–0.90) and 0.82 (0.78–0.86), respectively. The remaining markers (HOXA7, HOXD8, ZNF808) achieved AUCs greater than or equal to 0.94 (Fig. 3b). Collectively, this suggested that the 7 DMRs had excellent performance and merited further investigation.

**Table 2:** The 7 DMRs for lung cancer diagnosis

Region	Gene symbol	Gene name	Feature type
chr17: 46,655,603–46,655,750	HOXB4	Homeobox protein Hox-B4	shore
chr17: 80,943,984–80,944,093	B3GNTL1	UDP-GlcNAc:betaGal beta-1,3-N-acetylglucosaminyltransferase like 1	opensea
chr19: 53,038,958–53,039,010	ZNF808	zinc finger protein 808	shore
chr2: 176,993,563–176,993,743	HOXD8	homeobox D8	island
chr2: 182,322,423–182,322,574	ITGA4	integrin subunit alpha 4	island
chr5: 40,681,077–40,681,250	PTGER4	prostaglandin E receptor 4	island
chr7: 27,195,684–27,195,794	HOXA7	homeobox A7	island

### Evaluation of the accuracy of Diagnostic model based on tissues

**Fig. 4:**
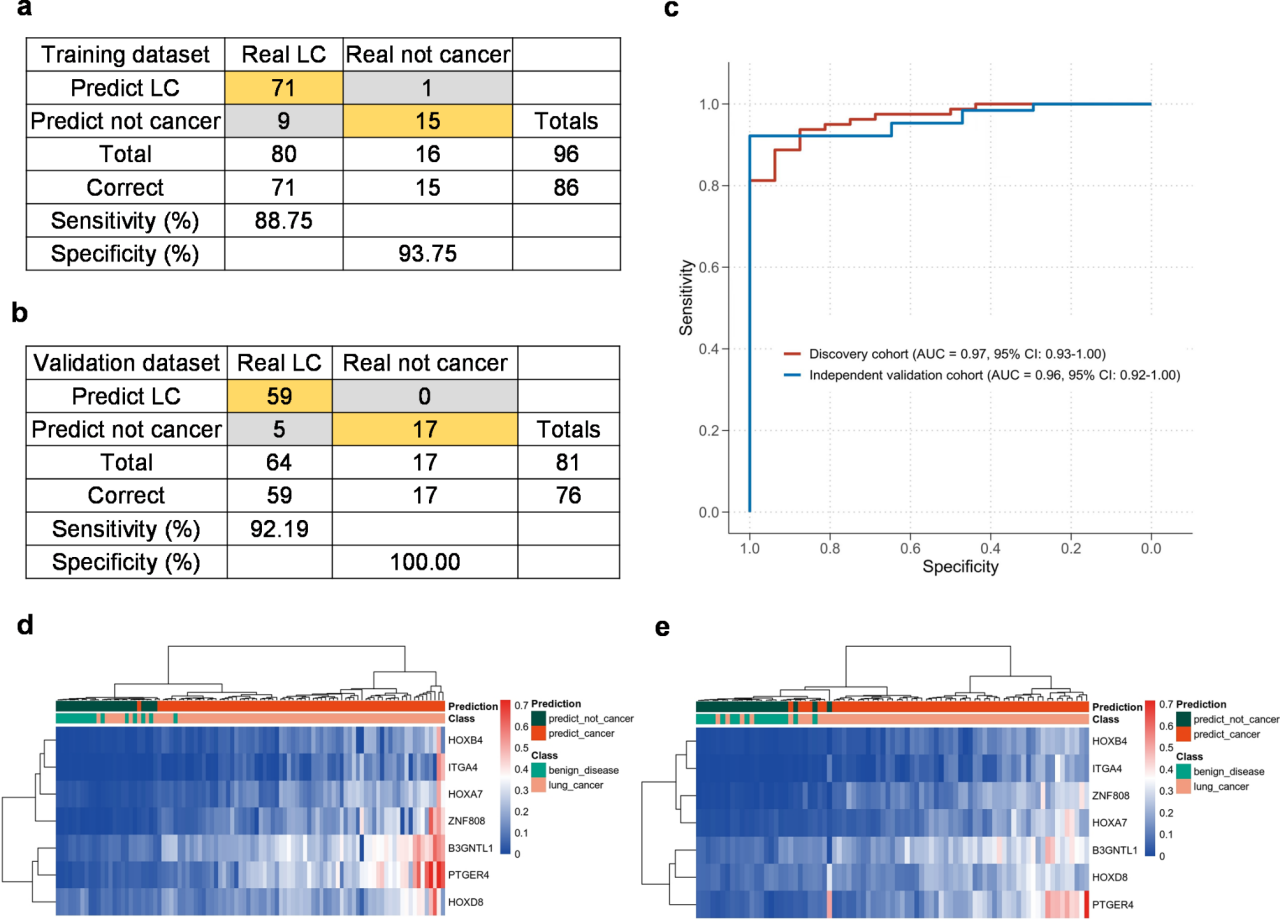
**Diagnostic performance of 7-DMR model in tissues. (a-b)** Confusion tables of binary results of the 7-DMR model in the training **(a)** and validation data sets **(b)**. **(c)** The representative ROC curves of 7-DMR model in lung cancer and benign nodule tissues in both discovery and validation cohorts. **(d-e)** Unsupervised hierarchical clustering of seven methylation markers for 7-DMR model in the training **(d)** and validation data sets **(e)** for tissues. LC: lung cancer

Next, we built a diagnostic model based on the panel of seven DMRs, namely 7-DMR model, using a training set of 80 lung cancer and 16 benign lung disease tissues. Accuracy of model in tissues was tested through the validation set of 64 lung cancers and 17 benign diseases. Our model achieved an AUC of 0.97 (0.93-1.00), sensitivity of 0.89 (0.82–0.95), specificity of 0.94 (0.89–0.97), and accuracy of 0.90 (0.84–0.96) in the discovery cohort and an AUC of 0.96 (0.92-1.00), sensitivity of 0.92 (0.86–0.98), specificity of 1.00 (1.00–1.00), and accuracy of 0.94 (0.89–0.99) in the independent validation cohort (Fig. 4a-c; Table 3). Unsupervised hierarchical clustering of these 7 markers was able to distinguish lung cancers from benign lung diseases with high specificity and sensitivity (Fig. 4d-e).

**Table 3:** The 7-DMR Model Performance Metrics, Values presented as: Mean, (95% C.I.)

Performance metric	Discovery cohort (n = 96)	Independent validation cohort (n = 81)	Plasma cohort (n = 106)
AUC	0.97 (0.93-1.00)	0.96 (0.92-1.00)	0.94 (0.86-1.00)
Sensitivity	0.89 (0.82–0.95)	0.92 (0.86–0.98)	0.81 (0.73–0.88)
Specificity	0.94 (0.89–0.99)	1.00 (1.00–1.00)	0.98 (0.95-1.00)
Accuracy	0.90 (0.84–0.96)	0.94 (0.89–0.99)	0.93 (0.89–0.98)
NPV	0.63 (0.53–0.72)	0.77 (0.68–0.86)	0.94 (0.89–0.99)
PPV	0.99 (0.96-1.00)	1.00 (1.00–1.00)	0.91 (0.86–0.97)

### Evaluation of the accuracy of Diagnostic model in plasma

**Fig. 5:**
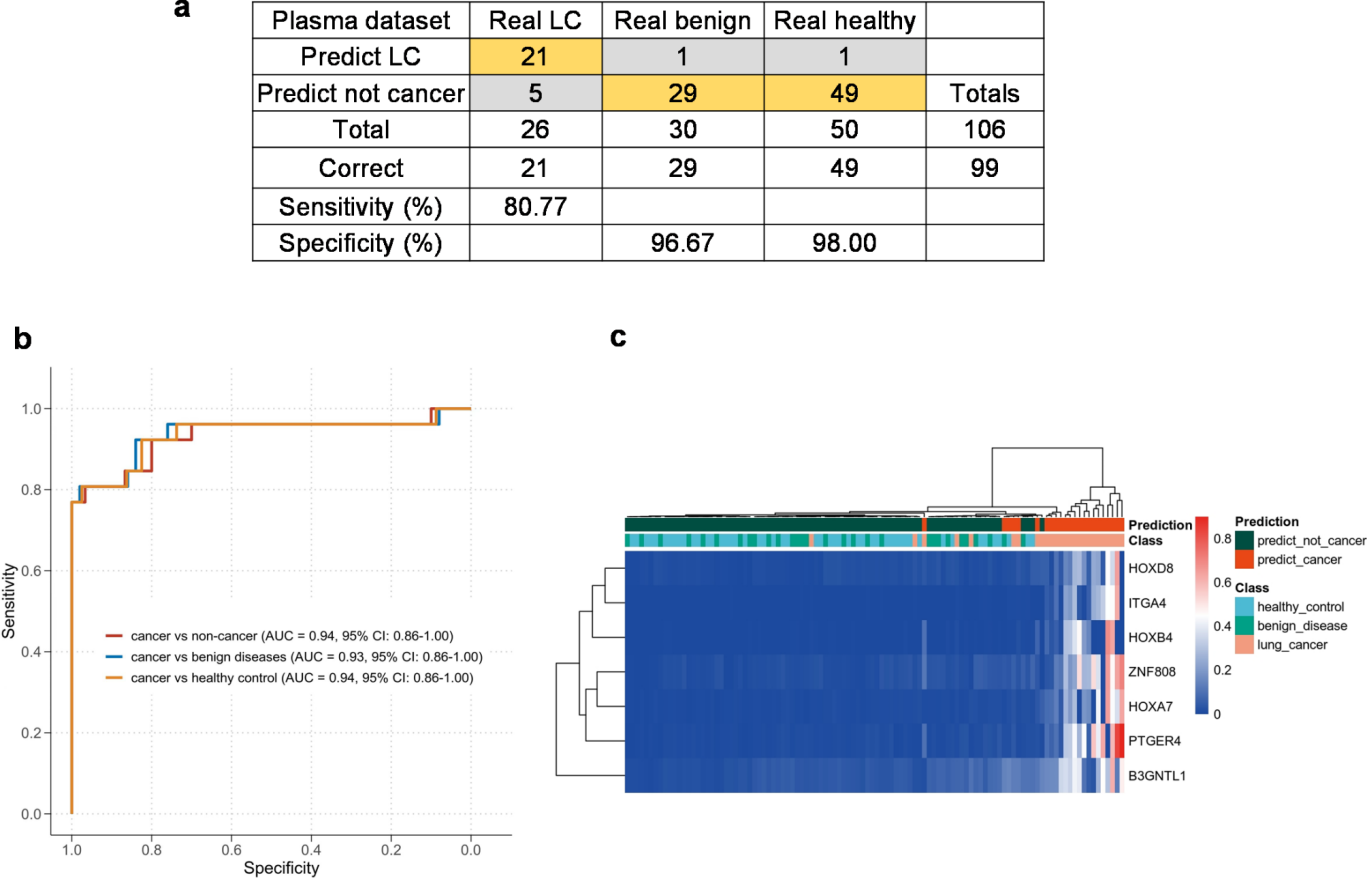
**Diagnostic performance of 7-DMR model in plasmas. (a)** Confusion tables of binary results of the 7-DMR model in plasma. **(b)** The representative ROC curves of 7-DMR model in plasmas of lung cancer, benign disease, and healthy control. **(c)** Unsupervised hierarchical clustering of seven methylation markers for 7-DMR model in plasmas.

Ideal biomarkers are expected to be detected non-invasively in biological fluids, we further tested the accuracy of 7-DMR model in 106 plasma samples. Consistent with the performance in tissue samples, the 7-DMR model achieved AUCs of 0.93 (0.86-1.00) in lung cancers vs. benign diseases, and 0.94 (0.86-1.00) in lung cancers vs. healthy controls. Incorporating the benign diseases and healthy controls as non-cancer group, the 7-DMR model still maintained stable diagnostic performance with an AUC of 0.94 (0.86-1.00), sensitivity of 0.81 (0.73–0.88), specificity of 0.98 (0.95-1.00), and accuracy of 0.93 (0.89–0.98) (Fig. 5a-b; Table 3). The precise diagnostic capability of the seven DMRs was also confirmed by the unsupervised hierarchical clustering among lung cancer, benign disease, and healthy control (Fig. 5c).

## Discussion

At present, the incidence of lung cancer and other cancers has risen sharply [23]. Although traditional pathological examination is still the gold standard for the diagnosis of various tumors, an accurate, non-invasive, and rapid diagnostic test is urgently needed in clinical practice. Numerous efforts have been devoted to searching for effective biomarkers. As aberrant DNA methylation patterns have been identified in lung cancer, DNA methylation biomarkers have been intensively investigated as potential diagnostic markers to detect early-stage lung cancer [24]. As early as 2005, Schmiemann V et al. discovered the abnormal methylation level of APC, p16 (INK4a), and RASSF1A genes in lung cancer patients, and proposed using methylation biomarkers for early diagnosis of lung cancer [16]. Recent research using prospectively and pre-diagnostic peripheral collected blood samples, a readily accessible sample source, is expected to provide valuable predictive marker data [25].

In this study, we systematically analyzed the methylation data of lung cancer. By comparing the methylation profiles of tissue samples between lung cancer and benign lung disease, we identified seven unique alterations in methylation that could function as promising biomarkers for early diagnosis of lung cancer. Each marker could accurately distinguish lung cancers from normal control. A diagnostic model of lung cancer constructed by the panel of 7 DMRs achieved a sensitivity of 92.2% and accuracy of 93.8%. The performance of the diagnostic model was evaluated in a set of plasma samples and well-discriminated results were also obtained. The abnormal expression of the DMRs related genes in lung cancer was confirmed by the TCGA gene expression data. The above data revealed that the 7 DMRs may play significant roles in development and progression of lung cancer that could be promising candidates for the development of diagnostic biomarkers in early-stage lung cancer.

### Strengths and limitations

Ideal diagnostic biomarkers are expected to be highly sensitive, specific to lung cancer, and non-invasively detectable at the early stage. We tested the seven biomarkers in a small set of plasma samples, and it showed superior diagnostic performance, indicating that the seven DMRs could be potentially applied as biomarkers in clinical practices. But the sample size was limited in this study and next we are going to recruit more participants to verify the generalization of the model. The development of lung cancer is a complex process involving multiple genetic, epigenetic, and protein expression alterations. Constructing predictive models using methylation biomarkers merely to assess the diagnosis of lung cancer may be inadequate. In the future, we will consider combining multi-omics such as radiomics, DNA fragmentation patterns, and proteomic biomarkers to further improve the predictive performance. Furthermore, intensively investigation on the functions of the targeted genes is necessary to clearly elucidate the molecular events occurring in the lung cancer development and progression.

## Conclusion

In summary, we identified seven novel lung cancer specific methylation markers that was able to discriminate the lung cancer from non-lung cancer. Our study demonstrates that the 7-DMR panel is of great value in the diagnosis of early-stage lung cancer, and thus may be potentially utilized as a noninvasive risk assessment tool for lung cancer before resection surgery.

## Electronic supplementary material

Below is the link to the electronic supplementary material.

Supplementary Material 1

## Data Availability

AnchorDx Medical Co., Ltd provides access to the study protocol, the statistical analysis plan, the clinical study report, and all individual participant data except genetic data with academic researchers. Access is provided after a proposal has been approved by an Independent Review Committee identified for this purpose and after receipt of a signed Data Use Agreement. Proposals should be directed to jianbingfan1115@smu.edu.cn.

## Abbreviations

DMR: Differential Methylation Region
mRMR: minimum Redundancy and Maximum Relevance
cfDNA: cell-free DNA
DMG: Differentially Methylated Gene
AUC: Area Under the Curve
ROC: Receiver Operating Characteristic
CYFRA 21 − 1: cytokeratin 19 fragment
CEA: carcinoembryonic antigen
NSE: neuron specific enolase
SCC-Ag: squamous cell carcinoma antigen
FFPE: Formalin-Fixed Paraffin Embedded
WHO: World Health Organization
DMC: Differentially Methylated CpG
KEGG: Kyoto Encyclopedia of Genes and Genomes
GO: Gene Ontology
HOXB4: Homeobox protein Hox-B4
B3GNTL1: UDP-GlcNAc:betaGal beta-1,3-N-acetylglucosaminyltransferase like 1
ZNF808: Zinc Finger Protein 808
HOXD8: Homeobox D8
ITGA4: Integrin Subunit Alpha 4
PTGER4: Prostaglandin E receptor 4
HOXA7: Homeobox A7
LUAD: Lung Adenocarcinoma
LUSC: Lung Squamous Cell Carcinoma
CI: Confidence Interval
GO: Gene Ontology
PPV: Positive Predictive Value
NPV: Negative Predictive Value
LC: lung cancer.
