# Supplementary material for: Accurate detection of early-stage lung cancer using a panel of circulating cell-free DNA methylation biomarkers
Source: Biomark Res. 2023 Apr 26;11:45. doi: 10.1186/s40364-023-00486-5 (PMC10134678; doi:10.1186/s40364-023-00486-5)
Supplement: Supplementary file 1 — Supplementary Material 1 [file 40364_2023_486_MOESM1_ESM.docx]

**Accurate detection of early-stage lung cancer using a panel of circulating cell-free DNA methylation biomarkers**

Shuo Hu, Jinsheng Tao, Minhua Peng, Zhujia Ye, Zhiwei Chen, Haisheng Chen, Haifeng Yu, Bo Wang, Jian-Bing Fan, Bin Ni.

**sMethods.**

**Figure S1.** The KEGG pathway analysis of the corresponding genes of hypermethylated and hypomethylated sites.

**Figure S2.** Gene ontology functional analysis of the corresponding genes of hypermethylated and hypomethylated sites.

**sReference.**

**sMethods**

**Tissue** **sample** **processing and targeted cell-free DNA methylation sequencing**

Five to ten-micron sections of FFPE tissue samples from patients were collected. Full details of DNA fragmentation, bisulfite conversion and targeted methylation sequencing were described previously ^1^. In brief, gDNA from 198 samples were fragmented into ~200 bp (peak size) by a M220 Focused-ultrasonicator (Covaris, Inc.) following the manufacturer’s instructions. 50 ng of purified fragmented gDNA was used for the following bisulfite conversion step. Bisulfite conversion was performed using EZ DNA Methylation-Lightning Kit (Zymo Research, Cat# D5031) according to the manufacturer’s protocol. AnchorIRIS library preparation technology was applied to targeted methylation analysis. AnchorIRIS pre-hybridization library construction was performed by AnchorDx EpiVisio Methylation Library Prep Kit (AnchorDx, Cat# A0UX00019) and AnchorDx EpiVisio Indexing PCR Kit (AnchorDx, Cat# A2DX00025). Target enrichment was performed using AnchorDx EpiVisio Target Enrichment Kit (AnchorDx, Cat# A0UX00031). A custom-made lung cancer methylation panel (LC Panel), which includes 9,307 differentially methylated regions (DMRs) covering 96,236 CpG sites identified by comparison of 28 lung cancer tissue samples with paired adjacent normal tissue samples, was utilized in present study ^2^. After probe hybridization, the resulted libraries were sequenced at the 2×150-bp paired-end mode on the NovaSeq 6000 platform.

**Blood sample processing and targeted cell-free DNA methylation sequencing**

10 mL of peripheral blood was collected from eligible patients 1-3 days prior to surgical operation in Streck Cell-free DNA BCT tubes (Streck, Cat# 218962) according to the manufacturer’s instructions and transported to AnchorDx’s certified molecular diagnostic laboratory within 48 hours. Plasma was separated immediately from the whole blood samples upon reception using a two-step centrifugation protocol. First, whole blood samples were centrifuged at 1,600g for 10 min at 4˚C, and the 1st batch of supernatants was transferred into 1.5 mL tubes. Next, the 1st batch of supernatants was centrifuged at 16,000g for 10 mins at 4˚C, and the 2nd batch of supernatants was transferred into new 1.5 mL tubes and immediately stored at -80°C until use. cfDNA isolation was carried out using the MagMAX Cell-Free DNA Isolation Kit (Thermo Fisher Scientific, Cat# A29319) according to the manufacturer’s protocol. The concentration of cfDNA was determined by Qubit dsDNA HS Assay Kit (Thermo Fisher Scientific, Cat# Q32854), and the quality was verified by an Agilent 2100 Bioanalyzer using the Agilent High Sensitivity DNA Kit (Agilent Technologies, catalog 5067-4626).

Full details of targeted cfDNA methylation sequencing were described previously ^2^. In brief, bisulfite conversion was carried out with the EZ DNA Methylation-Lightning Kit (Zymo Research, catalog D5031) following the manufacturer’s protocol. 10 ng cfDNA was used for AnchorIRISTM pre-hybridization library construction using the AnchorDx EpiVisioTM Methylation Library Prep Kit (AnchorDx, Cat# A0UX00019) and the AnchorDx EpiVisioTM Indexing PCR Kit (AnchorDx, Cat# A2DX00025). The amplified pre-hybridization libraries were subsequently purified using Agencourt AMPure XP Magnetic Beads (Beckman Coulter, Cat# A63882), and the concentration was measured by the Qubit dsDNA HS Assay Kit. Pre-hybridization libraries containing > 400 ng of DNA were considered qualified for the subsequent target enrichment. Target enrichment was conducted by the AnchorDx EpiVisioTM Target Enrichment Kit (AnchorDx, Cat# A0UX00031). The custom-made methylation panel used in this study, which consisted of 9,307 preselected differentially methylated regions enriched for lung-cancer specific methylations, was described above. After hybridization, particular fragments of the DNA libraries bound with biotinylated probes were pulled down by Dynabeads M270 streptavidin beads (Thermo Fisher Scientific, Cat# 65306). The enriched libraries were then amplified by P5 and P7 primers using KAPA HiFi HotStart Ready Mix (KAPA Biosystems, Cat# KK2602), and the amplified libraries were then purified by Agencourt AMPure XP Magnetic Beads. The final libraries were quantified by Qubit dsDNA HS Assay Kit before being sequenced on the NovaSeq 6000 System (Illumina).

**Sequencing data analysis**

Sequencing data analysis was performed as previously reported ^2^. Briefly, the sequencing quality of raw reads was evaluated by FastQC software (v0.11.4). Sequencing adapters and 3′ low-quality bases were trimmed from raw sequencing reads using fastp (v0.19.6). After quality control, clean reads were mapped to the C-to-T in silico converted hg19 reference genome using Bismark (v0.17.0). Aligned reads were then evaluated by Picard (v2.5.0) for metrics that measured the performance of target capture-based bisulfite sequencing assays (http://broadinstitute.github.io/picard). The biases of specific motifs or GC-enriched regions were excluded. After the preliminary analysis, the coverage for each CpG site were calculated. The CpG sites with coverage less than 30× were excluded from downstream analysis.

**Figure S1.** **The KEGG pathway analysis of the corresponding genes of hypermethylated and hypomethylated sites.**

**Figure S2.** **Gene ontology functional analysis of the corresponding genes of hypermethylated and hypomethylated sites**. (**A**) Cellular component; (**B**) Molecular function; (**C**) Biological processes terms.

**eReference**

1. Liang W, Zhao Y, Huang W, et al. Non-invasive diagnosis of early-stage lung cancer using high-throughput targeted DNA methylation sequencing of circulating tumor DNA (ctDNA). *Theranostics*. 2019;9(7):2056-2070. doi:10.7150/thno.28119

2. Liang W, Chen Z, Li C, et al. Accurate diagnosis of pulmonary nodules using a noninvasive DNA methylation test. *J Clin Invest*. May 17 2021;131(10)doi:10.1172/JCI145973
